# Structure-based Design Targeted at LOX-1, a Receptor for Oxidized Low-Density Lipoprotein

**DOI:** 10.1038/srep16740

**Published:** 2015-11-18

**Authors:** Shraddha Thakkar, Xianwei Wang, Magomed Khaidakov, Yao Dai, Kuppan Gokulan, Jawahar L. Mehta, Kottayil I. Varughese

**Affiliations:** 1 Department of Physiology and Biophysics College of Medicine, University of Arkansas for Medical Sciences, Little Rock, Arkansas, USA; 2 Department of Medicine, College of Medicine, University of Arkansas for Medical Sciences, Little Rock, Arkansas, USA

## Abstract

Atherosclerosis related cardiovascular diseases continue to be the primary cause of mortality in developed countries. The elevated level of low density lipoprotein (LDL) is generally considered to be the driver of atherosclerosis, but recent years have seen a shift in this perception in that the vascular plaque buildup is mainly caused by oxidized LDL (ox-LDL) rather than native-LDL. The scavenger receptor LOX-1 found in endothelial cells binds and internalizes ox-LDL which leads to the initiation of plaque formation in arteries. Using virtual screening techniques, we identified a few potential small molecule inhibitors of LOX-1 and tested their inhibitory potential using differential scanning fluorimetry and various cellular assays. Two of these molecules significantly reduced the uptake of ox-LDL by human endothelial cells, LOX-1 transcription and the activation of ERK1/2 and p38 MAPKs in human endothelial cells. In addition, these molecules suppressed ox-LDL-induced VCAM-1 expression and monocyte adhesion onto human endothelial cells demonstrating their therapeutic potential.

Atherosclerosis is a chronic progressive disease, and its clinical manifestations include coronary artery disease, cerebrovascular disease and peripheral arterial disease. The interaction between the lectin-like oxidized low-density lipoprotein receptor-1 (LOX-1) and oxidatively modified low-density lipoprotein (ox-LDL) plays a significant role in the pathobiology of atherosclerosis, as well as myocardial ischemia and hypertension^1^. Conditions leading to atherosclerosis, such as diabetes, hypertension and dyslipidemia, are associated with a global increase in inflammatory signaling and generation of reactive oxygen species (ROS), leading to enhancement of LDL oxidation. LOX-1 mediates the recognition and internalization of ox-LDL by vascular endothelial cells^2^. Of all known scavenger receptors, LOX-1 is the predominant scavenger receptor in vascular endothelial cell^3^,^4^. Recent studies show that blockade of LOX-1 by anti-sense oligo or antibody may reduce myocardial ischemic injury, genesis of hypertension and extent of atherosclerosis^5^,^6^,^7^,^8^. Hence, LOX-1 is an attractive target for the therapy of a number of cardiovascular disease states^9^.

LOX-1 is a transmembrane protein comprising four domains and the C-terminal domain is responsible for ox-LDL recognition^10^,^11^. The crystal structure analysis of the C-terminal domain of human LOX-1 (Fig. 1A,B) suggests that it exists as a homodimer with a central hydrophobic tunnel that extends through the entire molecule^12^,^13^.

Under oxidative stress, LDL undergoes changes making its surface electronegative. The most significant modification pertinent to LOX-1 interaction is the covalent attachment of a phospholipid moiety on the Lys side chains of apolipoprotein B-100, a component of LDL^13^,^14^,^15^. Modeling studies have shown that the phospholipid moiety fits well into the hydrophobic tunnel of LOX-1^16^. Binding measurements suggest that it ox-LDL binds to LOX-1 with high affinity. Additionally, mutations of certain residues present in the tunnel impair binding to ox-LDL, confirming the crucial role of the tunnel in ligand recognition and binding^16^. Another structural feature relevant to ox-LDL binding is called ‘basic spine’, and it is a linear arrangement of basic residues across the dimer surface (Fig. 1B)^13^,^15^. It has been proposed that these residues interact with a long helix of the apolipoprotein B protein of LDL^15^.

Inhibition of ox-LDL binding with LOX-1 can be achieved by either blocking the ‘basic spine’ or the tunnel, but a ligand can have more interactions in a tunnel than on the surface resulting in higher affinity and specificity. Therefore we searched for molecules that can occupy the tunnel. Some investigators have described substrate mimics or natural inhibitors that inhibit LOX-1 activity, albeit in large concentrations^17^,^18^,^19^,^20^,^21^,^22^. The present paper describes identification of chemical inhibitors of LOX-1 and their efficacy in blocking LOX-1 expression and activity in nanomolar quantities.

We used structure-based drug design (SBDD) techniques for finding inhibitors of LOX-1 that block the hydrophobic tunnel and prevent ox-LDL/LOX-1 interactions. Towards this goal, we explored a diverse chemical space provided by the ZINC data base without limiting ourselves to substrate analogues or natural products^23^. After identifying several lead molecules, we evaluated the activity of the top five candidates and observed that two of them exerted a highly significant inhibition.

## Results

**Figure 1:**
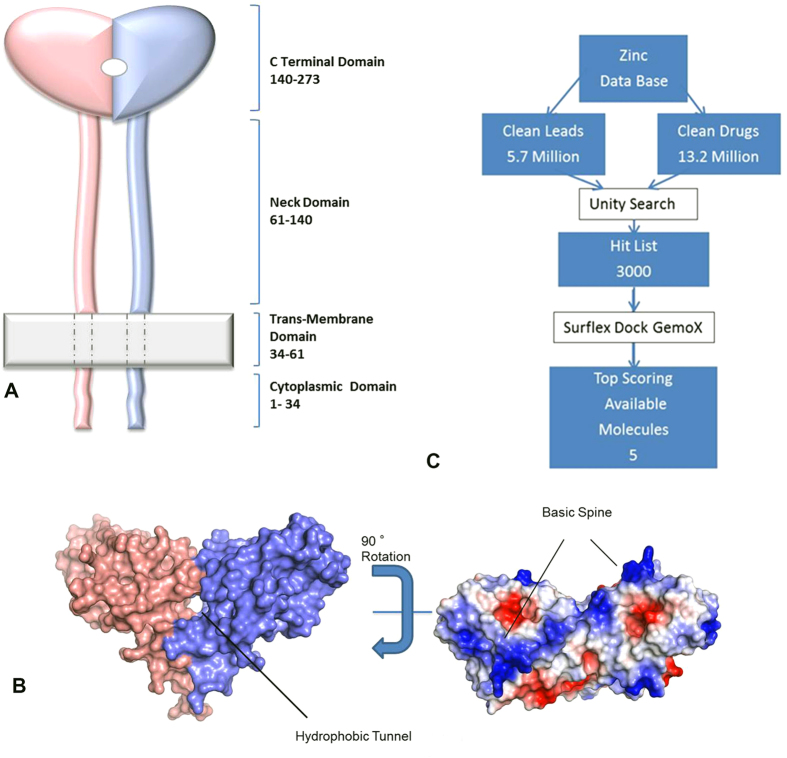
The structure of LOX-1 and its interaction with ox-LDL. (**A**) A cartoon showing the domain structure of LOX-1. LOX-1 is a transmembrane protein with 273 residues comprising 4 domains. The first 36 residues form a cytoplasmic tail, followed by a single transmembrane domain (21 residues), and an extracellular region comprising two domains. The first one (58–142) is predicted to be a coil, and the second (143–273) is a C-type lectin-like domain (CTLD) responsible for ox-LDL recognition and it exists as a disulfide-linked homodimer^12^,^13^. (**B**) Left panel: A view of the surface representation of the C-terminal domain exhibiting the central tunnel. Right panel: A rotated view of the surface showing ‘basic spine’, the linear arrangement of basic residues. (**C**) A flow chart showing steps involved in virtual screening.

**Figure 2:**
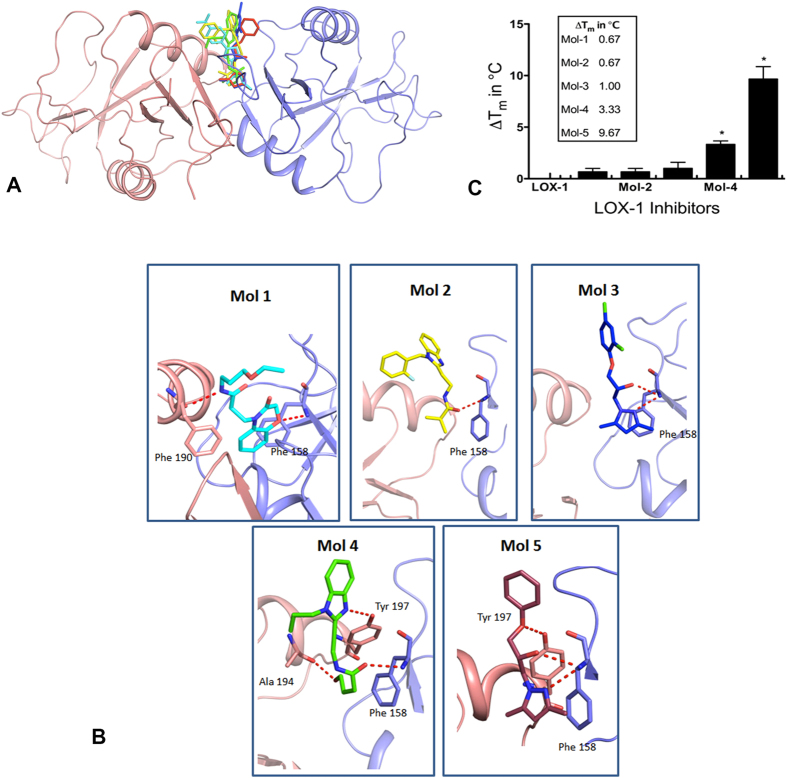
Docking of the 5 lead compounds and the thermal denaturation assay. (**A**) The figure depicts the docking of the 5 lead compounds in the binding pocket of LOX-1. LOX-1 is shown as a ribbon model with one monomer in magenta and the other in blue. The compounds are shown as stick models in separate colors. (**B**) Hydrogen bonding interactions of the compounds as they dock to LOX-1. (**C**) The measured thermal shifts (in Celsius) are depicted for all five molecules. The solutions contained LOX-1 protein at 4 μM concentration. The ligand concentrations were 50 μM.

We used virtual screening techniques to identify potential small-molecule inhibitors that bind tightly to LOX-1 to prevent ox-LDL/LOX-1 interaction. These calculations were performed with the software package SYBYL^24^ using high resolution crystal structures of LOX-1^12^ (PDB code 1YPQ). The large chemical libraries of the ZINC data base^23^ were used to extract potential inhibitors of LOX-1 and we picked five molecules with the highest scores for experimental investigations (Table 1) using the steps outlined in the flow chart (Fig. 1C). Figure 2A,B show simulated binding interactions in the tunnel of LOX-1 structure.

### Effect of different molecules on thermal stability of LOX-1 protein

The C-terminal domain of LOX-1 was expressed and purified using the protocols reported by Park *et al*.^12^ and the thermal shift assays^25^ were performed to test the binding of the computationally predicted molecules to the LOX-1 protein. The technique involves measuring thermal denaturation temperatures of the protein in the presence and absence of these molecules. A higher denaturation temperature in the presence of the inhibitor is indicative of strong interaction between the ligand and the protein. The five lead compounds were tested for their effect on the thermal stability of LOX-1 protein. Of five molecules tested Mol-5 and Mol-4 showed significant shifts (10 °C and 4 °C shifts, respectively) (Fig. 2C).

### Effect of the lead compounds on Ox-LDL Uptake by HUVECs

**Figure 3:**
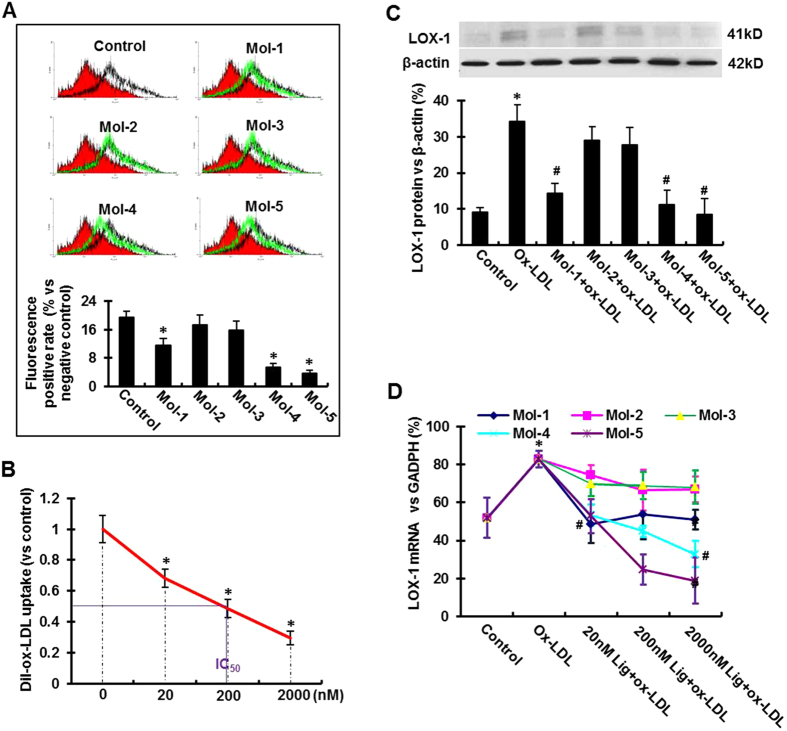
Inhibitory effect of the lead compounds on Dil-ox-LDL uptake by HUVECs and CHO cells (measured by flow cytometry) and the effect of the lead compounds on ox-LDL-induced LOX-1 mRNA and protein expression. (**A**) (Upper Panel) Flow cytometry measurements using HUVECs in the presence of the five lead compounds (Mol-1–Mol-5), each a at 200 nM concentration. (Lower Panel) The percentage of internalized ox-LDL. Graphs show data as mean (±SD); n = 4. *P < 0.01 or < 0.05 vs. control. (**B**) IC50 value for Mol-5. IC50 value (in nM) is based on inhibition of Dil-ox-LDL uptake in CHO cells overexpressing human LOX-1. Graphs show data as mean (±SD); n = 5. *P < 0.01 or < 0.05 vs. control. (**C**) Western blotting assay showing LOX-1 protein expression in HUVECs following incubation with 200 nM Mol-1 to Mol-5 for 30 min, and subsequently exposure to 5 μg/mL ox-LDL for 6 hours. (**D**) The variation of LOX-1 mRNA expression levels at different concentrations of the compounds following exposure of HUVECs to 5 μg/mL ox-LDL. The concentrations are indicated on the X-axis and the LOX-1 mRNA levels are shown on the Y-axis. The plots for all the five molecules are shown in separate colors. Graphs show data as mean (±SD); n = 3. *P < 0.01 vs. control; ^#^P < 0.01 vs. ox-LDL group.

We measured ox-LDL uptake by human umbilical vein endothelial cells (HUVECs) after exposure to 200 nM of each compound using flow cytometry. As shown in Fig. 3A, Mol-5 and Mol-4 significantly and potently inhibited ox-LDL uptake (P < 0.05), and Mol-1 also displayed a moderate inhibitory effect. Mol-2 and Mol-3 had no significant effect on ox-LDL uptake (P > 0.05). Overall, Mol-5 exhibited the maximum inhibitory effect on ox-LDL uptake by HUVECs (Fig. 3A). We also measured the dose response to modulation of ox-LDL uptake by Mol-5 (0, 20, 200 and 2000 nM) in CHO cells overexpressing human LOX-1. As shown in Fig. 3B, Mol-5 reduced ox-LDL uptake in a dose dependent manner, with IC_50_ value ~200 nM.

### Effect of the lead molecules on LOX-1 expression in HUVECs

Internalization of ox-LDL via binding to LOX-1 activates endothelial cells and upregulates the expression of LOX-1^2^. In this study, we tested if our lead molecules would inhibit LOX-1 expression. Accordingly, we measured LOX-1 protein expression in HUVECs by Western blotting. As shown in Fig. 3C, ox-LDL (5μg/ml), as expected, markedly increased LOX-1 protein expression (P < 0.01). Addition of Mol-1, Mol-4 and Mol-5, each at 200 nM concentration significantly reduced ox-LDL-induced LOX-1 protein expression (P < 0.05).

We also measured LOX-1 mRNA levels in HUVECs treated with ox-LDL in the absence or presence of the lead molecules at varying concentrations. As shown in Fig. 3D, exposure to ox-LDL (5 μg/ml for 6 h) significantly increased LOX-1 mRNA expression (P < 0.05), and Mol-1, Mo-4 and Mol-5 each significantly reduced ox-LDL-induced LOX-1 mRNA upregulation (P < 0.05).

### Effect of different molecules on the activation of MAP kinases in HUVECs

**Figure 4:**
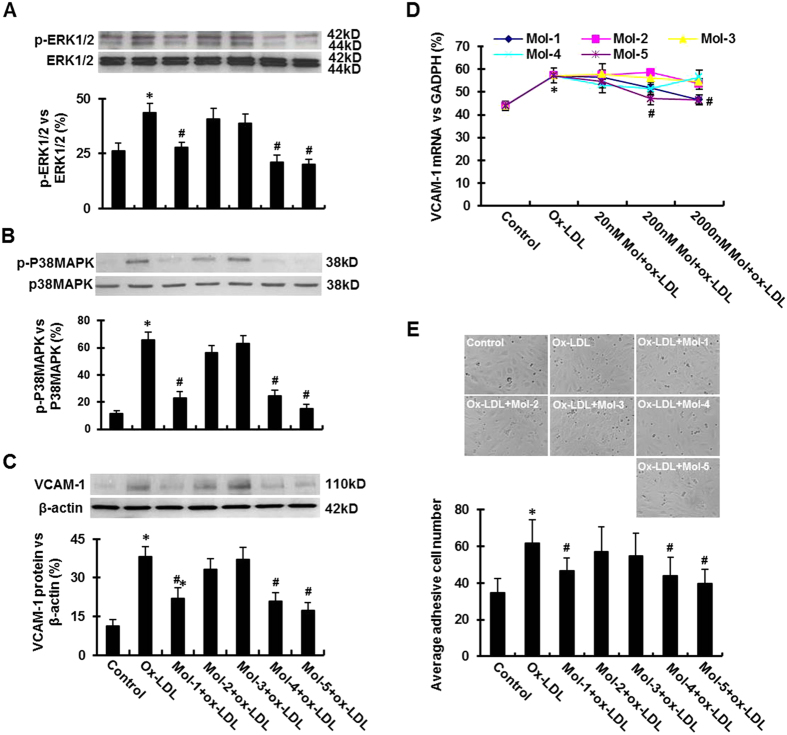
Effect of lead molecules on ox-LDL-induced the activation of MAPKs in HUVECs, VCAM-1 expression and monocyte adhesion onto HUVECs. (**A**) Western blotting assay shows total ERK1/2 and phospho-ERK1/2 expression in HUVECs following exposure to 200 nM of each compound and to 5 μg/mL ox-LDL. (**B**) Western blot analyses showing phospho-p38MAPK expression in HUVECs following exposure to 200 nM of each compound for 30 min, and subsequently exposed to 5 μg/mL ox-LDL for 6 hours. (**C**) Western blotting assay shows VCAM-1 protein expression in HUVECs following incubation with 200 nM Mol-1 to Mol5 for 30 min, and subsequently exposure to 5 μg/mL ox-LDL for 6 hours. (**D**) The variation of VCAM-1 mRNA expression levels with different concentrations of the led compounds. The concentrations are indicated on the X-axis and the VCAM-1 mRNA levels are shown on the Y-axis. The plots for all five molecules are shown in separate colors. (**E**) Exposure HUVECs to 5 μg/mL ox-LDL for 6 hours could significantly increase their adhesive ability to monocytes. Mol-1, Mol-4 and Mol-5 markedly inhibited ox-LDL-induced monocyte adhesion onto the HUVECs. Graphs show data as mean (±SD); n = 3 (for Western blot and PCR assays) or n = 20 (for monocyte adhesion assay). *P < 0.01 vs. control; ^#^P < 0.01 or < 0.05 vs. ox-LDL group.

To assess downstream effects of each candidate molecule, we measured ox-LDL induced expression and activation of ERK1/2 and P38MAPK in HUVECs in the absence or presence of each candidate molecule (200nM). As shown in Fig. 4A,B, Mol-1, MoL-4 and Mol-5 each significantly inhibited ox-LDL-induced activation of ERK1/2 and P38 MAPK (P < 0.01 or < 0.05). Of note, Mol-5 exhibited the maximum inhibition of activation of MAP kinases, and Mol-2 and Mol-3 had no significant effect on ox-LDL-induced activation of MAPKs. Importantly, unphosphorylated MAPKs were not affected by any of the candidate molecules.

### Effect of the lead molecules on VCAM-1 expression in HUVECs

It is known that the activation of LOX-1 by ox-LDL in endothelial cells stimulates the expression of leukocyte adhesion molecules^26^. In this study, we measured VCAM-1 expression in HUVECs in the presence of 5 μg/ml ox-LDL. As shown in Fig. 4C, exposure to ox-LDL substantially enhanced VCAM-1 protein expression, which was significantly reduced by Mol-1, MoL-4 and Mol-5 (each in 200 nM concentration) (P < 0.01 vs. ox-LDL alone). We also measured VCAM-1 mRNA expression in HUVECs exposed to 5 μg/mL ox-LDL for 6 hrs. As shown in Fig. 4D, ox-LDL increased VCAM-1 mRNA expression (P < 0.05), and Mol-4 and Mol-5 significantly reduced ox-LDL-mediated VCAM-1 mRNA upregulation (P < 0.05).

### Effect of the lead molecules on adhesion of monocytes to HUVECs

The ox-LDL-induced expression of adhesion molecules, such as VCAM-1, facilitates the adhesion of inflammatory cells, such as monocytes. In this study, ox-LDL treatment resulted in a dramatic increase in adhesion of monocytes to HUVECs, and Mol-1, MoL-4 and Mol-5 each at 200 nM concentration decreased the number of monocytes adherent to HUVECs (Fig. 4E; P < 0.01 or < 0.05).

## Discussion

The interaction of ox-LDL with LOX-1 on endothelial cells has several consequences, such as initiation of inflammation which is an early event in atherogenesis^27^,^28^,^29^. Ox-LDL binding to the cell surface receptor LOX-1 leads to the internalization of ox-LDL. The presence of ox-LDL in the subendothelial space promotes transformation of monocytes to macrophages and macrophages into foam cells, and subsequently initiates buildup of atherosclerotic plaque. The endothelial cells express LOX-1 several-fold more than other scavenger receptors^30^. Therefore, it has been proposed that LOX-1 inhibition may be useful in the treatment of conditions associated with LOX-1 over-expression^9^.

As described above, we identified 5 potential inhibitors of LOX-1 by virtual screening, and evaluated the activity of these 5 compounds using various assays. The differential scanning fluorimetry displayed significant thermal shift with Mol-5 and Mol-4 while it did not show measurable shifts with Mol-1, Mol-2 and Mol-3. Interestingly the cell-based assays show moderate activity of Mol-1, albeit lower than that of Mol-4 and Mol-5. Thus Mol-1 showed functional activity without inducing thermal shift. Although ligand binding generally enhances thermal stability, there are exceptions to this concept and sometimes binding can take place without change in thermal stability^31^. It is possible that Mol-1 binds to LOX-1 protein and alters activity without causing a significant shift in thermal stability.

We quantified the inhibitory effect of our lead compounds on the uptake of ox-LDL by HUVECs. Mol-5 showed the most potency as it reduced the uptake of ox-LDL by 80%, and Mol-4 was somewhat less potent as it reduced the uptake of ox-LDL by 65% (Fig. 3A), both at 200 nM concentration. Mol-1 reduced the uptake of ox-LDL by about 35%. Mol-2 and Mol-3 had no significant effect on ox-LDL uptake. Of note, Mol-5 also inhibited the uptake of ox-LDL by CHO cells transfected with human LOX-1 with an IC_50_ value of 200 nM (Fig. 3B). Thus, we confirmed the efficacy of Mol-5 in HUVECs as well as in CHO cells.

Ox-LDL interaction with LOX-1 stimulates the expression of LOX-1 in endothelial cells. Indeed, the exposure of HUVECs to ox-LDL in the present study resulted in ~60% increase in LOX-1 mRNA consistent with previous observations^2^. Inhibition of LOX-1 transcription by our lead compounds was assessed in three different concentrations (20, 200 and 2000 nM). We observed that both Mol-4 and Mol-5 reduced LOX-1 transcription at 200 nM concentration by 47% and 70%, respectively, reflecting the potency of these compounds. Mol-1, Mol-2 and Mol-3 had no significant effect on LOX-1 transcription.

The interaction of ox-LDL with LOX-1 in endothelial cells results in several down-stream effects, such as phosphorylation of MAPKs (including P38 and ERK1/2). P38 MAPK activation is thought to be pro-inflammatory, whereas ERK1/2 activation is related to cell proliferation, although this functional differentiation of function is not absolute. In the present study, ox-LDL had a dramatic effect on the phosphorylation of both P38 and ERK1/2 components of MAPK. As shown in Fig. 4A,B, Mol-5 reduced the level of phospho-P38 MAPK by 75%, Mol-4 and Mol-1 by around 60%. Mol-5 and Mol-4 also potently reduced ERK1/2 phosphorylation, and Mol-1 reduced ERK1/2 phosphorylation moderately, whereas Mol-2 and Mol-3 had no significant effect. Of note, ox-LDL did not affect the levels of unphosphorylated ERK1/2 or P38 MAPK. The lead compounds also did not affect the levels of unphosphorylated ERK1/2 or P38 MAPK.

Cell based assays revealed that the interaction of ox-LDL with LOX-1 leads to intense expression of VCAM-1 at both mRNA and protein levels. Consistent with previous data on ox-LDL internalization and intracellular signaling, Mol-4 and Mol-5 significantly inhibited the expression of VCAM-1 at both mRNA and protein levels. Treatment of HUVECs with ox-LDL increased the expression of VCAM-1 several-fold, and the lead molecules significantly reduced ox-LDL-induced expression of VCAM-1 (both mRNA and protein). As expected, VCAM-1 upregulation was associated with intense monocyte adhesion to HUVECs, and Mol-5, Mol-4 and Mol-1 each reduced monocyte adhesion significantly (by 35%, 30% and 25%, respectively) (Fig. 4E).

Since these molecules could potentially be used clinically, we estimated their cytotoxicity, and observed almost no toxicity until at least 2000 nM concentration (Supplementary material S1).

A number of new medications under development are biologics. Anti-hLOX-1 antibody has also been developed^32^ and it may prove to be effective in the treatment of atherosclerosis and related disorders. However, immunogenicity remains a limitation of antibody therapy^33^. Chemical inhibitors of LOX-1 may be equally effective as biologics without major side-effects.

As mentioned earlier, several laboratories have attempted to generate LOX-1 inhibitors focusing mainly on substrate mimics or natural products. Falconi *et al.*^17^ used modified phospholipids that bind to the tunnel in the LOX-1 molecule, and observed that their compounds prevented the uptake ox-LDL, but only at high concentrations. Sawamura and coworkers^20^ identified inhibitors from food extracts and found that procyanidins inhibited ox-LDL uptake in CHO cells expressing LOX-1; these compounds also prevented the uptake of ox-LDL. Yoshiizumi *et al.*^18^ reported on the inhibitory properties of sulfatid derivatives on scavenger receptors and observed that 2,4-bis(octadecanoylamino) benzenesulfonic acid prevented LDL from binding to macrophages. Using cell-based binding assays and molecular docking simulations, Biocca *et al.*^21^ recently showed that statins bind the hydrophobic tunnel and can inhibit ox-LDL binding to LOX-1, again in high concentrations. Similarly, aspirin also been reported to reduce ox-LDL mediated LOX-1 expression^34^. However, these studies do not show any direct interaction of aspirin with the LOX-1 receptor.

In summary, LOX-1 is a promising novel target of therapy of atherosclerosis and related disorders. Through virtual screening technique, we have identified several potential inhibitors of LOX-1, and two of these Mol-4 and Mol-5 were found to bind strongly with the LOX-1 protein. Cell based assays indicate that these molecules inhibit the uptake of ox-LDL and reduce downstream effects like MAPK activation. Interestingly, these compounds were found to significantly reduce the expression of adhesion molecules on endothelial cells and subsequent monocyte adhesion. We will be evaluating the pharmacokinetics (absorption, distribution, metabolism, and excretion) of these agents in near future.

## Materials and Methods

### Virtual screening to identify potential small-molecule inhibitors

In order to locate LOX-1 inhibitors, we screened two subsets of the of the ZINC data base^23^. The compounds in this data base obey the Lipinski rule, a rule of thumb for a compound to be orally active as a drug^35^. We carried out the search on two subsets namely, ‘clean leads’ and ‘clean drug-like’ that contain chemical data on 5,735,035 and 13,195,609 small molecules respectively. The notation “clean” indicates that the compounds in the subset do not contain aldehydes, thiols, or Michael acceptor sites. These compounds are prone to have toxicity issues^36^. The virtual screening procedures were carried out using the software package SYBYL^24^. In preparation for the docking calculations, the geometry of the protein molecule (PDB code 1YPQ) was optimized using energy minimization techniques after the addition of hydrogen atoms. The ZINC datasets were reformatted to Sybyl Line Notation (SLN) compatible with the software package SYBYL. The next step was the assignment of the binding pocket and it was done using the SiteID module in SYBYL, which identifies potential binding sites within the protein molecule^37^. The largest cavity obtained from this calculation was in agreement with the ligand binding site observed in the crystal structure 12 and also with the conclusions of the modeling studies of LOX-1 with oxidized phospholipid^16^.

Following the binding site identification, a MOLCAD surface was generated to incorporate all amino acids present on the binding surface within 5 Å. Additionally we identified a few potential hydrogen bond donors and acceptors in the active site as a part of the pharmacophore preparation. We performed the virtual screening using UNITY search module in SYBYL to identify molecules that could have the potential hydrogen bonding interactions and are capable to fit inside the MOLCAD surface generated in the binding pocket. This step served as a filter to generate a small subset of molecules that has potential to bind to LOX-1.

### *Scoring functions and the estimating the probability of binding*

The molecules belonging to the subset were docked to the binding site of LOX-1 using Suflex Dock-Gemox module SYBYL using default parameters. This procedure generates a protocol which is an idealized representation of the binding site that simulates the binding environment experienced by the ligand. The use of protomol expedites the docking process^38^. The docking analysis lists the C-Scores (consolidated scores) for the each molecule which is a measure of the goodness of fit. The C-Score function combines the binding score obtained from five different scoring algorithms namely, FlexX(Total Score), G_Score, PMF_Score, D_Score and ChemScore. These scores account for various interactions such as hydrogen bonding, electrostatic and hydrophobic interactions, entropy and solvation. C-Score 5 means that the binding score obtained from all five different scoring algorithms are above threshold and the ligand molecule has favorable interactions in the binding pocket^38^. While C score is a good indicator of binding, it is not a measure of the activity of the molecule. It should be pointed out that although all the molecules that we tested have a C score of 5, they do not have similar interactions at the binding pocket and each molecule has its unique mode of binding (Fig. 2B).

### Protein Expression and Purification

The C-terminal domain of LOX-1 was expressed by using the protocols reported earlier^12^. As this domain contains several disulfide bridges, it was co-expressed with DsbC, a Chaperone disulfide isomerase. The genes were custom synthesized by Genscript (Piscataway, NJ) using gene map described in Park *el al.*^12^. It encodes for 147 amino acids for the LOX-1 fragment (residues 136–273) and 236 amino acids for DsbC. The gene was incorporated into a pET15b vectors by Genscript. The vectors were then transformed into Dh5α cells for amplification and BL21 (DE3) E. coli (Novagen) cells for expression. The proteins were expressed by adding 0.4 mM isopropyl β-D-thiogalactoside in *E. coli* cells grown in LB medium containing kanamycin, tetracycline, and carbenicillin at 23 °C, with 250 rpm agitation, for 20 h. The protein was purified using a nickel-nitrilotriacetic acid column (Qiagen, Valencia, CA). And then, the protein samples were concentrated to 10 mg/ml.

### Thermal Shift Assay

LOX-1 protein was incubated with fluorescent dye Sypro Orange (Invitrogen/Life Technologies, Grand Island, NY) and the thermal unfolding of the LOX-1 protein was monitored by measuring the fluorescence using Bio-Rad Real Time PCR machine. We measured the melting temperatures in the absence and presence of the inhibitors 4 μM of LOX-1 protein was incubated with Sypro Orange and heated in stepwise increments of 0.5 °C per min from 25–99 °C. The wavelength for excitation was set to 490 and emission was set to 575 nm for Sypro Orange. Fluorescence was measured after every minute for each 0.5 °C increase.

### Culture of human umbilical vein endothelial cells (HUVECs)

The primary HUVECs were purchased from ATCC (Manassas, VA) and cultured in vascular cell basal medium with supplements (including 10 mL fetal bovine serum, 10mM L-glutamine, 0.5 μg hydrocortisone hemisuccinate, 25 μg ascorbic acid, 2.5 ng rhVEGF, 2.5 ng rhEGF, 2.5 ng rh FGF, 7.5 ng rhIGF-1 and 0.375 units heparin sulfate). Cells from passages 3 and 4 were used in this study.

### Dil-ox-LDL uptake measurement

HUVECs were plated into 6-well plates and cultured until the cells reached 70% confluence. The cells were exposed to 5 μg/mL Dil-ox-LDL for 30 min after treatment with 200 nM of each of 5 molecules for 30 min, and then washed with PBS for 3 times. After washing, the cells were digested with trypsin and washed with PBS, and suspended in 400 μL PBS, and analyzed with a flow-cytometer (Becton Dickinson, Mountain View, CA). For further confirm the inhibitory effects of Mol-5 on Dil-ox-LDL uptake, we also measured Dil-ox-LDL uptake by CHO cells after exposure to 0, 20, 200 and 200 nM Mol-5 for 30 min. The CHO cells were stably transfected with human LOX-1 cDNA.

### Western blotting

HUVECs were plated into 6-well cell culture plates. When the cells were reached 70% confluence, they were treated with 200 nM of each of 5 molecules for 30 min, and subsequently exposed to 5 μg/mL ox-LDL for 6 h. After washing with PBS for 2 times, the cells were treated with Cell Lysis Buffer (Promega, Madison, WI) with protease Inhibitor and phosphatase Inhibitor (Sigma-Aldrich, St. Louis, MO). The protein concentrations were measured by Bradford protein assay. Proteins (20 μg) were separated by electrophoresis in 12% precast SDS-PAGE gels, and were transferred onto Nitrocellulose Blotting Membrane (GE Healthcare Life Science, Piscataway, NJ). The membranes were then incubated with LOX-1, VCAM-1 (Abcam, Cambridge, MA), ERK1/2, phospho-ERK1/2, phospho-p38MAPK (Cell Signaling Technology, Inc., Danvers, MA), or β-actin (Santa Cruz Biotechnology, Inc., Dallas, Texas) primary antibodies (1:1000 ~ 1:2000) in blocking solution at 4 °C overnight. Following wash with TBS-1 for 3 times, the blots were incubated with HRP-conjugated secondary antibodies (1:10000) in blocking solution for 1 hour at room temperature. After 3 washes with PBS, the blots were treated with Western Blotting Luminol Reagents (Santa Cruz Biotechnology) for 3 min and then exposed to Blue Basic Autorad Film (GeneMate, Kaysville, UT). The protein bands were scanned by a Gel DocTM XR System (Bio-Rad, Hercules, CA), and the density of each band was quantified by Image J software. The relative expression of target proteins was calculated with comparison to β-actin bands.

### Real-time polymerase chain reaction (q-PCR)

HUVECs were plated into 12-well plates, and treated with 0, 20, 200 and 2000 nM of each of 5 molecules for 5 for 30 min, and subsequently exposed to 5 μg/mL ox-LDL for 6 h at 37 °C. Total RNA was isolated from the treated HUVECs by using a RNeasy Mini-Kit (Invitrogen/Life Technologies) according to the manufacturer’s instructions. Prior to using, RNA was treated with DNase I and diluted to100 ng/μL. 100 ng RNA was applied to synthesize cDNA with SuperScript III 1st Strand DNA Synthesis Kit. qPCR was performed using the Applied Biosystems Fast 7500HT real-time PCR system with a 20 μL reaction volume including 10 μL GoTaq qPCR Master Mix (Promega, Madison, WI), 100 ng of cDNA and 0.3 μM primers. Data was analyzed by using MX3000P software. Relative mRNA expression was quantified using the comparative threshold cycle (Ct) method. The sequences of primers used for PCR reaction have been described previously^30^.

### Monocyte adhesion assay

Human monocytes were isolated from blood from the heathy donors and cultured in DMEM supplemented with 10% FBS. HUVECs were plated into 6-well plates. When the cells reached 70–80% confluence, they were treated with 200 nM of each of 5 molecules for 30 min, and subsequently exposed to 5 μg/mL ox-LDL for 6 h. After washing with PBS, the medium was replaced, and then monocytes (5 × 105) were added to each well (4 wells/group) onto HUVECs. Thirty minutes later, the medium containing monocytes were aspired and the unattached monocytes were carefully washed out with PBS. Five fields of each well were randomly captured under a microscope. The number of adherent monocyte was counted and averaged.

### Cytotoxicity assay

HUVECs (1 × 10^3^/well) were plated into 96-well plates and cultured overnight. The medium was replaced after an overnight culture, and the cells were subsequently incubated with 20, 200 and 2000 nM of each candidate molecules (Mol-1, Mol-2, Mol-3, Mol-4, and Mol-5 diluted in ultrapure water) for 6.5 h. As control, cells were incubated with the same aliquots of ultrapure water. Cytotoxicity was tested using a Pierce LDH Cytotoxicity Assay Kit (Invitrogen/Life Technologies) as per manufacturer’s instructions. The LDH cytotoxicity was measured by absorbance of A490 minus A680.

### Statistical analysis

Statistical analysis was performed with SPSS 16.0 software. Data are presented as means and standard deviation (SD). Univariate comparisons of means were evaluated using one-way ANOVA with Tukey’s post-hoc adjustment for multiple comparisons. A P value < 0.05 was considered significant.

### No animal studies

The studies reported in this paper do not involve experiments on humans or other vertebrates.

## Additional Information

**How to cite this article**: Thakkar, S. *et al.* Structure-based Design Targeted at LOX-1, a Receptor for Oxidized Low-Density Lipoprotein. *Sci. Rep.* **5**, 16740; doi: 10.1038/srep16740 (2015).

## Supplementary Material

Supplementary Information

## Figures and Tables

**Table 1:**
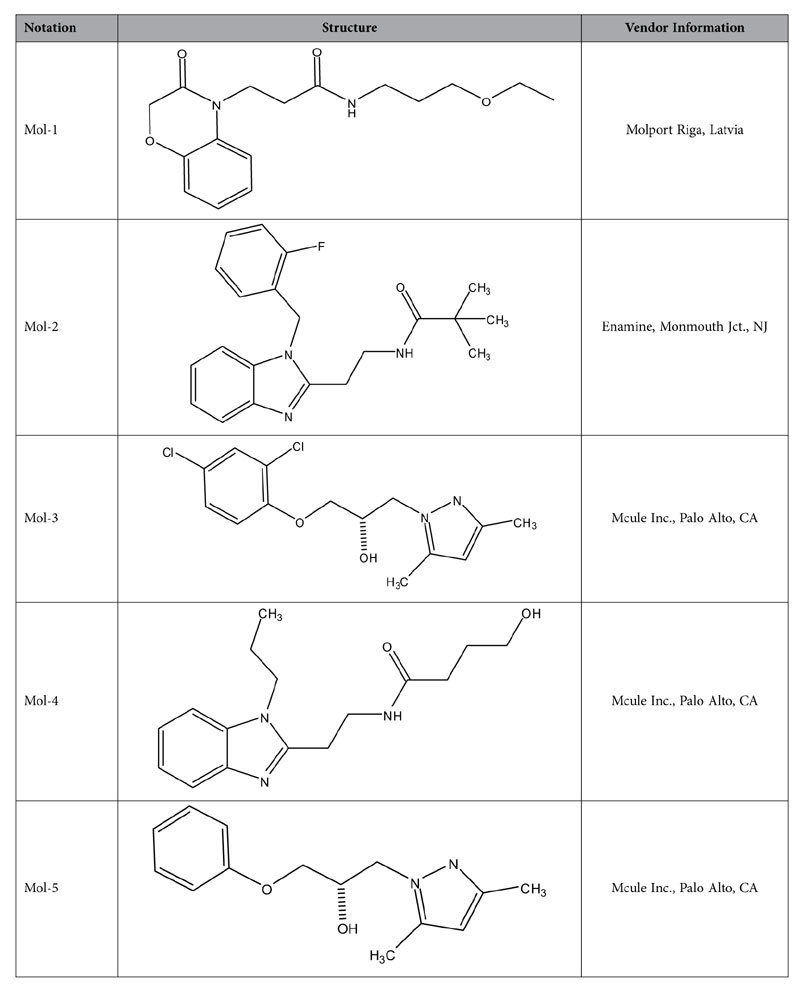
Lead Compounds.
