# Supplementary material for: Structure-based Design Targeted at LOX-1, a Receptor for Oxidized Low-Density Lipoprotein
Source: Sci Rep. 2015 Nov 18;5:16740. doi: 10.1038/srep16740 (PMC4649741; doi:10.1038/srep16740)
Supplement: Supplementary Information [file srep16740-s1.doc]

Supplementary Material:

**Structure-based Design Targeted at LOX-1, a Receptor for Oxidized Low-Density Lipoprotein**

Shraddha Thakkar^1#^, Xianwei Wang^2#^, Magomed Khaidakov^2^, Yao Dai^2^, Kuppan Gokulan^1^, Jawahar L. Mehta^1,2*^, Kottayil I. Varughese^1*^

^1^Department of Physiology and Biophysics, ^2^Department of Medicine , College of Medicine, University of Arkansas for Medical Sciences, Little Rock, Arkansas, USA.

LDH Cytotoxicity Data.


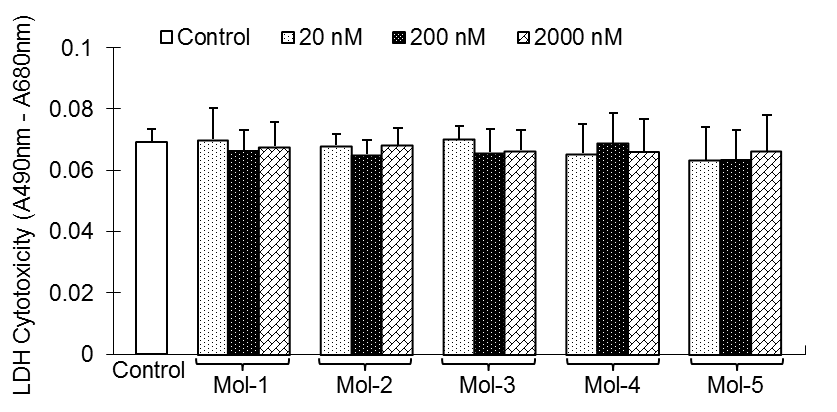


S1: Cytotoxicity was measured using a Pierce LDH Cytotoxicity Assay Kit after exposing HUVECs to three different concentrations (20nM, 200nM and 2000nM) of compounds for 6.5 hours. The control cells were treated with water without any compound. The cytotoxicity was measured as the absorbance at 490nm minus the absorbance at 680nm. Data are presented as mean ± SD; n = 4. There were no significant difference in cytotoxicity between control and experimental groups indicating the compounds do not any cell cytotoxicity even at 2000nM (P >0.05).
